# Imperfect Wound Healing Sets the Stage for Chronic Diseases

**DOI:** 10.1126/science.adp2974

**Published:** 2024-12-06

**Authors:** Paul Martin, Carlos Pardo-Pastor, R. Gisli Jenkins, Jody Rosenblatt

**Affiliations:** * School of Biochemistry, University of Bristol, Department of Medicine and Life Sciences, Universitat Pompeu Fabra; ^ Laboratory of Molecular Physiology, Department of Medicine and Life Sciences, Universitat Pompeu Fabra; † Margaret Turner Warwick Centre for Fibrosing Lung Disease, National Heart & Lung Institute, NIHR Imperial Biomedical Research Centre, Imperial College London; $ The Randall and Cancer Centres King’s College London; £ The Francis Crick Institute

## Abstract

Although the age of the genome gave us much insight about how our organs fail with disease, it also suggested that diseases do not arise from mutations alone; rather, they develop as we age. Here, we examine how wound healing might act to ignite disease. Wound healing works well when we are younger, repairing damage from accidents, environmental assaults, and battles with pathogens. Yet, with age and accumulation of mutations and tissue damage, the repair process can go off the rails, leading to inflammation, fibrosis, and neoplastic signalling. Here, we discuss healthy wound responses and how our bodies might misappropriate these pathways in disease. Although we focus predominantly on epithelial based (lung and skin) diseases, similar pathways might operate in cardiac, muscle, and neuronal diseases.

## Introduction

For over 160 years, the concept of cancer being linked to aberrant wound healing has ebbed and flowed with enough evidence to ensure the hypothesis was never completely rejected. Originally, Virchow noted inflammatory infiltrates in tumours (1), with Haddow proposing over-healing as a tumour initiation mechanism (2) and Dvorak later referred to tumours as ‘wounds that do not heal’ (3). Additionally, the finding that viral infections lead to cancer (4), not only supported the idea that infection and inflammation could contribute to cancer but was pivotal to identifying the first oncogenes. Discovery of viral transforming oncogenes then led to the discovery of cellular proto-oncogenes that become overactive or mutated in cancers (5). These findings together contributed to a picture where wound healing, viral infection, and inflammation, coupled with genetic alterations could promote neoplastic growth. Since then, diverse discoveries in genetics, epigenetics, immune-therapy, and metabolism have woven themselves into this fabric, encouraging us to reconsider aberrant wound healing with new perspectives.

A recent slew of papers suggests that while oncogenic mutations act as necessary drivers of cancer, they are not sufficient for tumour formation or progression (6–10). As we age, ‘normal’ tissue becomes riddled with oncogenic mutations that only ignite into tumours from triggers that promote wounds such as surgeries, smoking, pollution, infections, or chronic inflammation. This suggests a picture where homeostatic cell turnover can withstand oncogenic mutations but when challenged by tissue repair signals, lead instead to aberrant, pathological responses. Perhaps it is not surprising that common carcinoma drivers and hallmarks also act during wound healing to drive epithelial cell division and migration, such as the EGFR/RAS/MAPK/FGF/TGF-ß (11). The concept of aberrant wound healing is not restricted to cancer, as a growing number of other chronic diseases have many hallmarks of wound healing gone awry.

In this review, we examine how wound healing occurs in healthy, normal tissue and then explore how different diseases may result from defective wound responses. While these concepts are not novel, we hope to bring a fresh perspective to our current focus on mutations and precision medicine towards one that also recognizes generalized tissue responses. By instead holistically considering disease within the scope of our bodies trying to do their best to persistently heal wounds—but in the backdrop of defective wound healing pathways—we may gain a better overarching picture into a wide array of disease aetiologies. Our hope is that by understanding the chronology of these different diseases, we may identify key upstream events that spark the disease state and identify new ways to prevent them. Here, we examine how defective wound healing from trauma as well as environmental damage and pathogens contributes to inflammatory diseases, fibrosis, and cancer initiation and progression.

## Healthy Wound Healing

All living organisms have evolved strategies for repairing their cells and tissues after accident or conflict. In some organisms, such as axolotls and even teleost fish, this repair can be near-perfect and is then described as regenerative, but in adult mammals the best repair is always imperfect and leaves behind a permanent collagenous scar. Even this imperfect healing is complex and involves the coordinated actions of many different tissues and cell lineages. It requires tight orchestration of cell migration, proliferation, matrix deposition and contraction, followed by remodelling, alongside inflammation and angiogenesis (12).

### Phases of wound healing

**Figure 1:**
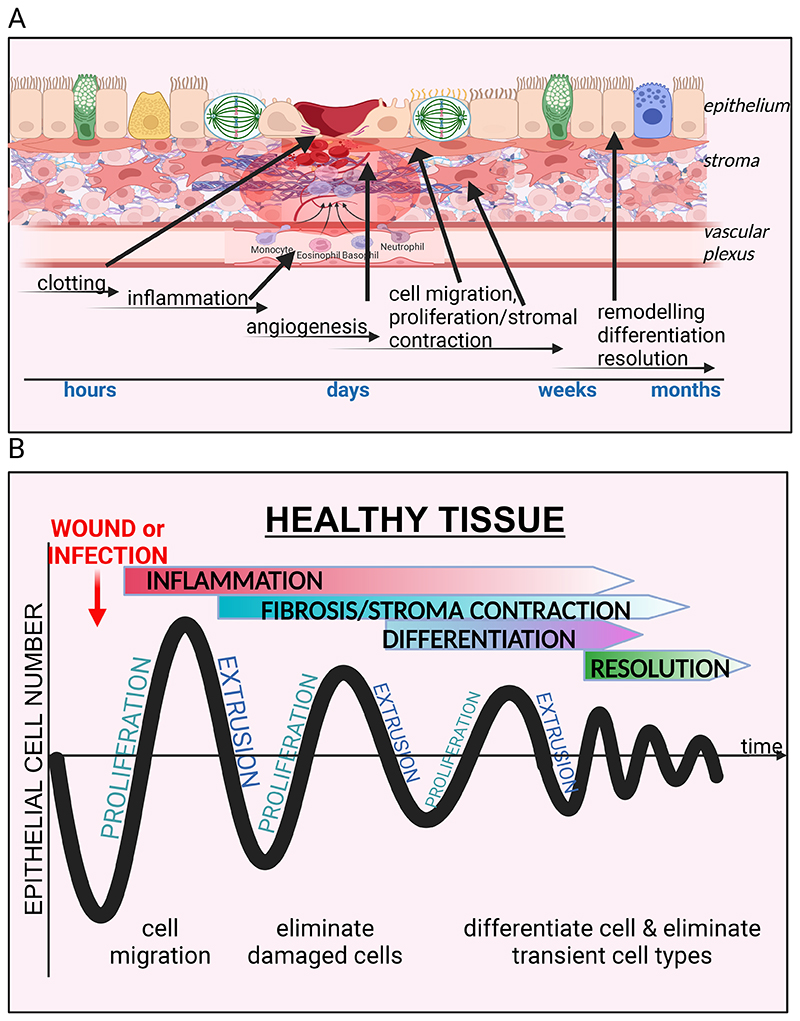
Healthy wound healing response. (A) Schematic of wound healing response in a healthy tissue with broad timeline below. Here, a break in the epithelium by damage or infection initiates a clot to stem bleeding and gaps and signal an inflammatory response to eliminate damaged cells and pathogens. The gap is repaired by epithelial cell migration and proliferation, followed by rounds of elimination of damaged cells by extrusion and differentiation into different specialized epithelial cell types. Simultaneously, the underlying stroma contracts to reduce the surface area and secretes extracellular matrix to reinforce and repair the damaged area, and recruit neurons, adipocytes, and vasculature to regenerate the wound site to an approximation of its former state. (B) Schematic graph of epithelial cell numbers over time, as cells proliferate, extrude, differentiate, to repair the site.

Tissue repair, is easiest visualised and most fully characterised in skin repair but is similar for all tissues of the body, proceeding through a coordinated series of phases (Fig. 1A). The process is classically divided into clot formation, inflammation, new tissue formation (including re-epithelialisation and granulation tissue formation and contraction), and finally, tissue remodelling, and resolution. If the tissue damage is in a barrier layer, such as the skin or an epithelial organ, then this barrier breach must be rapidly sealed to prevent fluid loss and inhibit invasion by pathogens. If the vasculature is in any way damaged, then platelets (and blood) will spill from the ruptured vessel and their activation, together with activation of the coagulation cascade, will lead to formation of a mesh-like clot to seal the defect. Platelet degranulation at this early-stage supplies some of the signals that will initiate aspects of the repair process, including the recruitment of inflammatory cells. Acute inflammation is classically associated with redness, heat, swelling, and pain. These symptoms typically result from activation of cytokines, histamines, prostaglandins, and bradykinins that dilate blood vessels and increase their permeability to allow extravasation of phagocytes and adaptive immune cells (lymphocytes), as well as non-immune cells like fibroblasts. Once the threats have been extinguished, acute inflammation resolves by reducing pro-inflammatory signals and leukocyte recruitment, typically through IL-10 and TGF-ß upregulation (13, 14), without which wounds cannot resolve (15). Importantly, phagocytosis of neutrophils by macrophages is a key step in resolution, as cells that are not silently removed can trigger the adaptive immune system (13, 16).

### Sealing the epithelial layer

Any damaged epithelial layer repairs by re-epithelialisation, directed by leading edge cell migration and a zone of proliferation back from the leading edge (17–19). After the epidermal wound edges have met and fused, and migration ceases, cell proliferation spreads into the just-sealed central zone and then ceases, presumably by density contact inhibition, the molecular mechanisms of which are still not clear.

Several hundred genes are switched on in the advancing epidermal wound edge cells – some requiring epigenetic unsilencing (20). While some of these are linked to cell division, most reflect fundamental roles required by epithelial cells to traverse through the wound terrain and seal the wound, including, for example, switching on of “resilience” pathways to cope with the harsh inflammatory environment, transient tethering to matrix, proteolysis of matrix and other debris, and the loosening of cell-cell junctions, which may extend back many tens of cells from the leading edge (21–23). These changes are often described as resembling a partial epithelial-to-mesenchymal transition (EMT). Notably, tissues act co-operatively to repair a wound; for example, as re-epithelisation is occurring, the underlying wound connective tissue is actively contracting (see later) to reduce the exposed surface that the epithelium must cover.

### Wounding triggers a cascade of “damage” signals to recruit inflammatory cells

Any damage to the skin barrier results in exposure to pathogen-associated molecular patterns (PAMPs) from invading microbes (24) as well as the rapid liberation of diverse damage signals, generally termed Damage Associated Molecular Patterns (DAMPs), by the cells at the wound edge. Recognition of PAMPs and DAMPs by immune cell receptors like TLRs and RAGE acts both to direct the migration of inflammatory cells towards the local wound site, and to trigger transcription of pro-inflammatory cytokines (25).

Neutrophils are the first immune cells to arrive at the wound, and their subsequent timely resolution and/or death at the wound site is crucial to prevent chronic inflammation. Some neutrophils are passively drawn into the wound from leaking wound vessels, but the majority actively extravasate from the bloodstream and migrate towards the wound site, attracted by the damage released signals. Once at the wound site, the primary role of neutrophils is to kill microbes through a combination of targeted Reactive Oxygen Species (ROS) release and the spewing out of chromatin to form neutrophil extracellular traps (NETs) (26). In healthy wound healing, after the neutrophilic microbicidal job is done, most neutrophils die and are cleared by macrophage phagocytosis (efferocytosis), which, in turn, impacts macrophage phenotype and function.

Macrophages recruit to wounds following the initial neutrophil inrush. Once at the wound site, macrophages phagocytose extracellular matrix (ECM) and cell debris, including “spent” neutrophils. Macrophages dynamically change from pro- to anti-inflammatory phenotypes, crucial for orchestrating the various phases of healing. Early arriving M1 macrophages are cytotoxic and microbicidal and later they transition to an M2 or ‘alternatively activated macrophage’ phenotype, which also alters their metabolism (27).

### Macrophages orchestrate healthy repair by signalling to other cell lineages at the wound site

At the wound site, macrophages regulate a wide range of cell behaviours, in various cells including fibroblasts, endothelial cells, adipocytes, and melanocytes. These influences are dynamic and change over the duration of healing to help reform the wound site to its prior state. Macrophages are fundamental in signalling fibroblasts to transition into myofibroblasts and deposit collagen in aberrant ways, resulting in fibrosis at the repairing wound site (28). Wnts, TGFβs, PDGF, and Relm impact fibroblasts responses (29, 30), which in turn, upregulate various scar effectors, including osteopontin that contributes to fibrotic response and lysyl hydroxylase-2, which cross-links collagen fibrils, making them resistant to remodelling (29, 30). TGFβ, in particular, is key in fibroblast collagen deposition and contraction in fibrosis (31). TGFβ isoforms may be critical in disease, as TGFβ1 can be pro-fibrotic whereas TGFβ3 may be anti-fibrotic (32, 33). Much less is understood about what shuts down this cycle of collagen deposition and fibroblast contraction in a healthily healing wound. If these signals persist aberrantly, then wound pathology results.

What is now clear is that different fibroblast populations respond in diverse ways: in the skin, deeper reticular and hypodermal fibroblasts are more scar prone and less able than superficial papillary fibroblasts to contribute to appendage regeneration (34). Gross anatomical locations of the body also have different predispositions to scarring, with sites like the back being very prone and the mouth resistant (35, 36). These differences may be due to a combination of additional molecular heterogeneity of effector fibroblasts, potentially linked to developmental origin and differing mechanical forces within different body sites (37).

All tissue injury sites experience a significant neoangiogenic response, with massive sprouting of the local vasculature to accommodate the increased metabolic demands of the repair process, also seen at sites of tumour growth (see later). In skin wounds this response is observed as a pink granulation tissue during the period of healing and subsequently resolves when the repair is complete. Macrophages initiate angiogenesis by physically shunting inhibitory neutrophils away from vessel tips before closely associating with the new angiogenic sprouts, supplying them with VEGFA. Knockdown of macrophages at early stages inhibits wound angiogenesis, whereas later knockdown, or forcing of macrophages to stay pro-inflammatory, prevents resolution or pruning of wound vessels (38, 39).

Neutrophils and macrophages are the most studied and predominate in the acute inflammatory response during healthy wound healing, but other immune cells also contribute. Mast cells are drawn to sites of tissue damage and remain present throughout the repair process but may act redundantly, since several knockdown studies have not led to repair defects (40). Sentinel-like γδT cells within the skin from embryonic stages become activated by local damage and release FGFs and IGF that are both trophic and mitogenic for keratinocytes at the wound edge (41).

With this general understanding of how wound repair normally works, we now consider how different steps in this process can go awry to promote disease, particularly if the repair mechanisms are repeatedly called upon. Given that wound healing at its best is imperfect, multiple rounds of repair will lead to accumulated defects, especially with age as our machines that do this repair suffer rust and breakdowns. The built-in errors in wound repair can be further amplified by accumulating mutations and mechanical changes associated with simple wear and tear on one’s body. While we focus mainly on epithelial repair, especially in the skin and lung, similar reiterated wound responses may be key drivers in other pathologies such as heart disease, other forms of fibrosis, and arthritis.

## Aberrant Inflammatory Responses

### Acute severe inflammatory responses

In large part, healthy wound healing is called upon relatively rarely within any given tissue, and although efficient, with further rounds and age, healing efficiency wanes (42). While acute inflammation is critical to recover from wounding and infection, as described above, there are circumstances where acute inflammation can overwhelm repair responses through excess damage or ineffective repair mechanisms that can lead to severe organ damage and death. A good example of this is in acute lung injury (ALI), which has many causes, and was dramatically witnessed during the covid-19 pandemic. Following ALI, epithelial cells die, damaging basement membranes and endothelial structures. Repair activates cell migration, proliferation, and trans-differentiation of epithelia, as described above. The degree of initial injury determines the outcome which can lead to death in 30-50% (43), and persistent fibrosis in about 11% of cases(44). Typically, residual damage does not improve or worsen after 6 months, although it may set the stage for progressive fibrosis in those with a genetic predisposition (13, 14, 16). These catastrophic events lead to considerable loss of epithelial cells that must be replaced. As vertebrates age their ability to recover from acute lung injury diminishes, as observed in both murine models of ALI (45) and in Covid-19 induced ALI, due to immunosenescence, hyper-inflammatory responses and other undetermined mechanisms (46–48).

### Chronic Inflammatory Responses

By contrast, long-term inflammation can be less obvious, yet equally hard to manage. Central to chronic inflammation management is knowing its cause. While the current strategy is to develop drugs that target genetic mutations and dysregulation, genetics appear to play a very limited role in chronic inflammatory diseases, aside from mutations in the Human Leukocyte Antigen system (49). A study following 210 twins revealed that 77% of all measured parameters of immune variation depended on environmental, rather than genetic factors (50). What then, are the environmental factors that impact inflammation? While age is the single largest factor, other contributors are annoyingly diffuse, ranging from obesity, poor sleep, stress, chronic infections, microbiome components, and the ever-increasing presence of microplastics (51–54). One common experience for patients with inflammatory diseases is flare-ups between periods of relative calm, suggesting preventing the exacerbations could be beneficial.

As ‘an ounce of prevention is worth a pound of cure’, it is important to identify central causes of chronic inflammation and exacerbations. The first essential step to innate immunity is the epithelial barrier, as infection and inflammation are impossible without first breaching it (55). We propose that chronic inflammation may stem further upstream at the level of epithelia. Epithelia, coated with a sugary glycocalyx, not only comprise our skin, but also mucosal membranes that line our organs. Their ability to secrete squalene, mucus, lipids, and antimicrobials help protect against pathogen invasion. Additionally, epithelia can prevent inflammation by physically shoving out cells infested with toxins, pathogens, or other damage by seamlessly extruding them (56–58). Given that chronic inflammation could stem from a defective epithelial barrier, the current approach of treating only the inflammation will only partially mitigate symptoms of a more central problem—ongoing wound healing.

### Continuous and aberrant wound healing

**Fig. 2:**
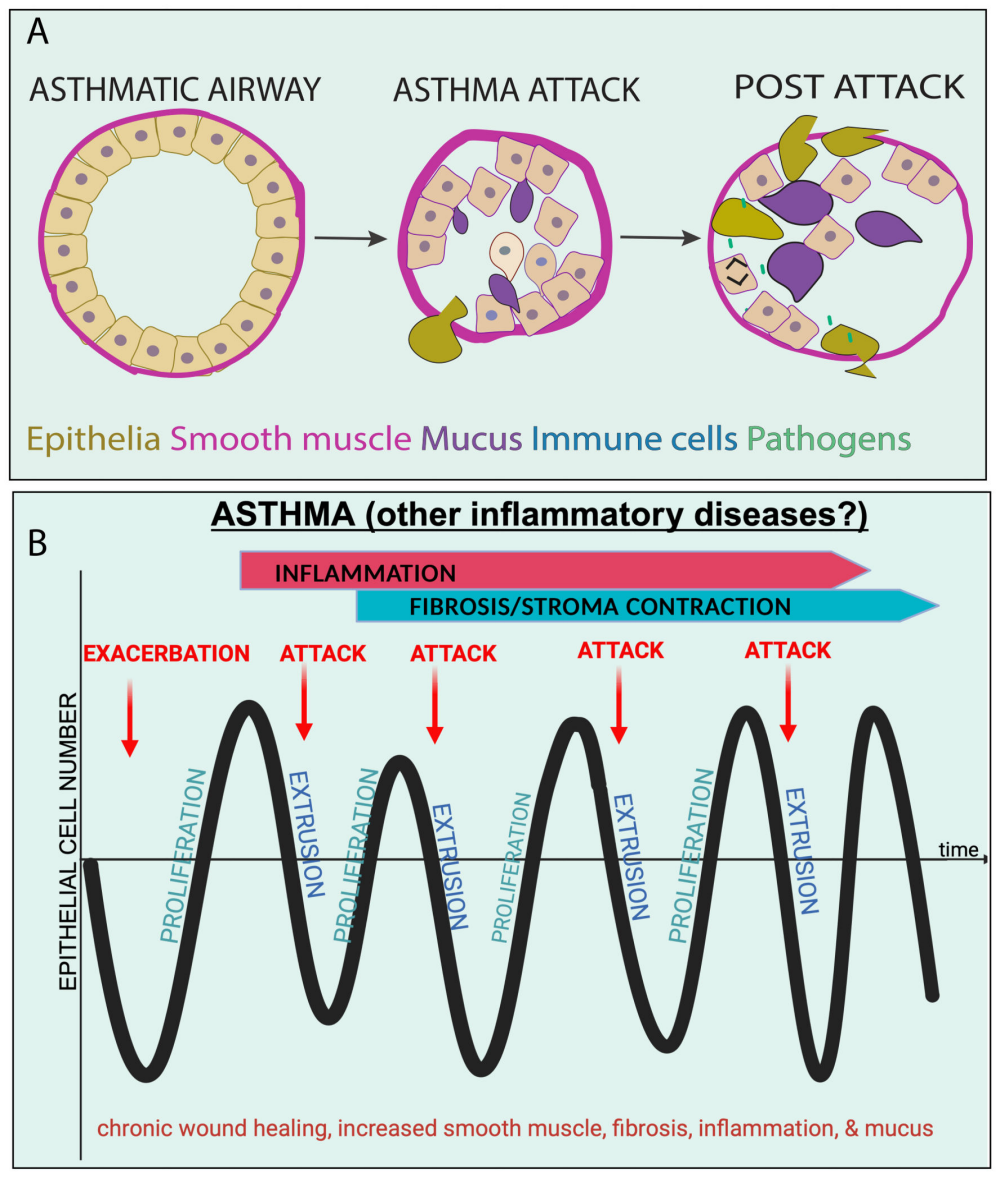
Continuous wound healing in asthma. (A) Schematic of an asthmatic airway before and after a bronchoconstriction. Amplified smooth muscle becomes hyperresponsive causing tight constriction to asthma triggers, which leads to excess crowding of epithelia and so much extrusion that it essentially creates a wound and excess mucus secretion. The wounding from an asthma attack then causes the inflammatory period and hypersensitivity to infections that cause more attacks. (B) Schematic of continuous wound healing from ongoing asthma attacks, driving cycles of extrusion and proliferation, where mucus amplification can be protective for wounds but, in excess, cause complications for patients.

In asthma, continued epithelial wounding results from repetitive mechanical attacks. While it might not be intuitive that airway constriction leads to wounding, the impetus for these studies derived from previous work identifying that mechanics control epithelial cell turnover to maintain constant cell densities. Here, the physical crowding from too many cells causes some cells to seamlessly squeeze out of the layer by a process called cell extrusion (56, 59). Typically, during homeostasis, crowded live cells extrude to later die; however, apoptosis, infection, and oncogenic transformation can also trigger cell extrusion (56–58, 60). Alternatively, when there are too few cells, the stretch forces they experience trigger some cells to rapidly divide to return to homeostatic densities (61). Remarkably, a stretch-activated channel, Piezo1, senses both opposing forces to trigger opposing responses. During normal homeostatic turnover, approximately 1.6-fold crowding elicits extrusion (59). However, the pathological crowding of an asthma attack causes so much extrusion that it essentially wounds the airway epithelial barrier, stimulating inflammation (62) (Fig. 2A). Interestingly, the crowding of the attack also causes mass secretion of mucus in the primary airways, which also hampers breathing. While reversing bronchoconstriction with the long-acting beta agonists does not abrogate either the wounding or mucus secretion, generic stretch-activated channel inhibitors do, suggesting a new approach to preventing long term asthma symptoms.

Preventing excess extrusion during airway contraction not only prevents inflammation but, by preventing airway epithelial damage, may prevent progression into phenotypes like chronic obstructive pulmonary disease (COPD) or lung cancer. Patients experiencing continuous asthma attacks suffer constant wounding of their airway epithelia that not only drives inflammation but airway remodelling from infiltration of macrophages and fibroblasts, which can amplify smooth muscle hypertrophy and remodelling, and contribute to the fibrosis associated with asthma and COPD (63). Indeed, the degree of airway wounding is directly linked to COPD severity (64, 65). It is possible that damage to epithelial linings may act similarly in promoting other inflammatory diseases like inflammatory bowel disease (IBD) and irritable bowel syndrome (IBS), since gastrointestinal infections, ulceration from anti-inflammatories, and cramping are classic exacerbators (66, 67).

We propose that many inflammatory diseases have sporadic, environmental causes that may effectively cause cyclical wound healing (Fig. 2B). It is not clear why some people rapidly resolve these wounds and others do not. It may be that affected individuals harbour mutations in genes not necessary for normal cellular turnover but critical in response to wound healing. In other cases, it might be due to poor luck of an individual getting a series of infections or continuous exposure to irritants, pollutants, or allergens. Continuous re-injury could contribute to the activation and hyperplasia of underlying smooth muscle that causes contraction, and mucus secretion. While in some contexts these are positive wound repair responses that would reduce the surface area not covered by epithelia, it could create a feed-forward cycle that instead drives disease states. If this hypothesis is correct, it could lead to an alternative approach to contraction-associated inflammatory diseases, where preventing epithelial damage, upstream of inflammation, would prevent the remodelling that drives the hyper-responsive disease state.

## Progressive Fibrosis

**Fig. 3:**
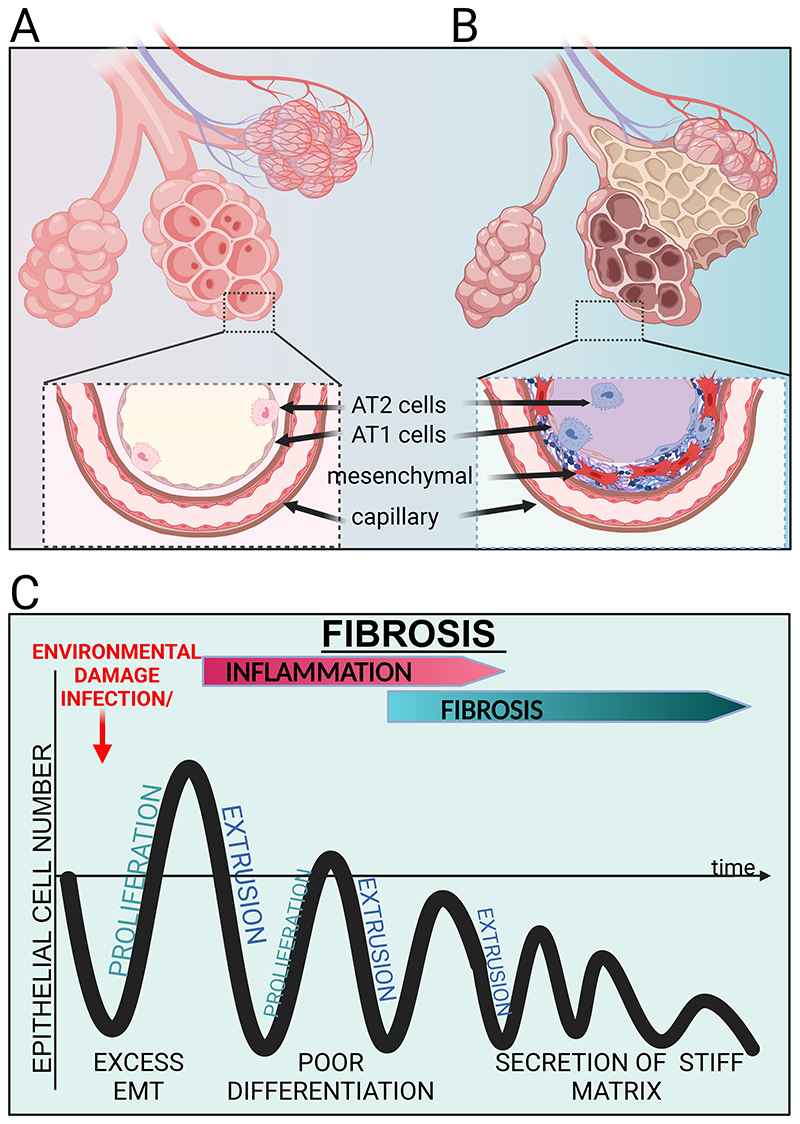
Excess differentiation and poor proliferation wound response in fibrosis. Schematics of a healthy (A) and fibrotic (B) alveolar sacs with capillaries beneath. In (A) healthy numbers of AT2 precursor cells and AT1 cells are lost in fibrosis (B) with cells differentiating and accumulating mesenchymal cells within the interstitial space between the alveolar sacs and capillaries. Here, TGF-ß and increasingly stiff matrix can act as a feed-forward cycle to transdifferentiate epithelial cells into fibroblasts that secrete too much matrix and stiffen it. (C) Schematic graph suggesting a poor response to wounding triggers a loss of epithelial cells over time, with increased mesenchymal cells that secrete and bundle stiff matrix.

It has long been considered that the inflammation and continuous aberrant wound healing leads to the development of progressive organ fibrosis (known as scarring in the skin) and has been the mainstay of therapeutic intervention for many years. Fibrosis is associated with aging and metabolic abnormalities that occur in response to a range of known and unknown genetic and environmental factors. Understanding shared pathogenic mechanisms, especially in early disease, may identify shared disease clusters that could respond to lifestyle modification or repurposed available therapies. Multiple physical changes occur in organs during fibrosis. As fibrosis progresses, excess secreted extracellular matrix (ECM) compresses and obliterates surrounding capillaries, which reduces blood flow and oxygen delivery, diminishes tissue water mobility, and reduces oxygen consumption (Fig. 3A&B). Altogether, these responses induce profibrotic stimuli that drive a fibrogenic feedback loop. As with chronic inflammation, fibrosis likely reflects a primitive protective mechanism, in the case of fibrosis, to wall off organs from infection, as seen in tuberculous granulomas, evolutionarily selected for in the pre-antibiotic era (68).

### Pathways to Fibrosis

Fibrosis can take months to years to materialise. Fibrosis occurs in several different settings which, depending on genetics, determine the long-term consequences: 1) In response to acute severe epithelial damage, such as in covid-19 induced acute lung injury, where epithelial cells die, resulting in damaged basement membranes and endothelial structures; 2) Chronic or recurrent low-grade exposure to uncleared infections or toxic substances can also lead to fibrosis, such as tuberculosis or schistosomiasis in lung or intestine, and alcohol in hepatic fibrogenesis. In such cases, removal of the insult or infection before structural organ damage can completely resolve the fibrosis. 3) In people suffering from autoimmune diseases such as systemic sclerosis, or Crohn’s disease, persisting immune responses may lead to fibrosis from chronic inflammation stimulating a fibrotic response. In these cases, treating the underlying inflammatory response will limit fibrosis and organ destruction. 4) A subgroup of people who develop fibrosis across organs with minimal or no apparent injury can result from rare variants in genes that lead to cellular injury, such as the unfolded protein response (alpha-1-antytrypsin deficiencies in liver fibrosis or surfactant proteinopathies such as Hermanski Pudlack Syndrome in lung fibrosis) or in premature aging syndromes, such as telomeropathies (Dyskeratosis Congenita) (69).

Fibrosis can occur in any organ, but the precise phenotype depends on the nature of the insult and the tissue response to it. This has been well characterised in the lung where the estimated genetic liability of sporadic pulmonary fibrosis is only 15% (70), suggesting a predominant environmental contribution. Just as inhaled insults contribute to lung fibrosis (e.g. pollution and cigarette smoke) and ingested toxins (alcohol) to gastroenterological fibrosis, any injurious agents can affect any organ, as exemplified by the increased risk of Alzheimer’s Disease in patients with high PM2.5 exposure to (71). Genetics also affect outcome. Of the 17 common human variants associated with the increased IPF risk, MUC5B is distinct in its aberrant upregulation specifically in alveoli, where patients with a MUC5B minor allele have a better prognosis following COVID-dependent acute lung injury (72, 73) and protection from childhood bacterial infections (74). Why increased mucin 5B protein protects against infection and lung injury but increases fibrosis risk is unknown. One possibility is that increased mucin 5B could alter tissue mechanics, which, would in turn, affect alveolar cell extrusion and proliferation (61). As the genetic link to IPF is weak (75) and generic processes like premature senescence (76) and impaired repair (77) more relevant, we consider the role of wound healing in fibrosis initiation and progression.

### Dysregulated Wound Healing

Damaging agents that promote wound healing responses can also trigger fibrosis through profibrotic interleukins such as IL1B and IL6. Notably, anti-IL6 therapy has recently received approval for treating fibrosis (78). Furthermore, mechanosignalling pathways involved in wound healing such as TAZ and MRTF are critical drivers of a pro-fibrotic epithelial phenotype associated with fibrosis (79) and pirfenidone, recently approved to treat IPF, can inhibit MRTF (80). As mentioned previously, TGFβ is a key regulator for wound repair resolution, as its absence promotes ongoing inflammation (81). Intriguingly, TGFβ signalling plays important roles in IPF and cancer, depending on the context. TGFβ can prevent epithelial hyper-proliferation to act as a tumour suppressor yet can promote tumour progression by driving epithelia to mesenchymal (or plastic) transition (EMT) (82). Additionally, TGFβ activation can prevent inflammation and emphysema following lung injury (83), yet its amplification can promote tissue fibrosis in both the parenchyma (84) and airways (85). One possibility is that TGFβ promotes EMT of alveolar epithelial cells into mesenchymal cells (86) that in turn secrete and bundle ECM in the interstitium between the alveoli and vasculature (Fig. 3A&B). How could the same signal prevent fibrosis in one context and promote it in another? Current thinking suggests that mechanical differences could be the key. Lungs are highly dynamic organs which expand and contract with each breath, and these mechanics are crucial for homeostasis in both the developing (87) and adult lung (83). Yet, a faulty wound healing response to any of the above triggers could end up with an amplified resolution step leading to progressive loss of specialized epithelial cells as they transition into mesenchymal cells (Fig. 3C). Since fibrosis converts soft, spongy tissue into firmer, wood-like tissue, increasing stiffness might shift homeostatic TGFβ responses into profibrotic ones (88, 89) (Fig. 3C).

## Cancer

Both cancer and fibrosis results from uncontrolled cell proliferation and matrix deposition and spread that occurs in response to environmental injury and can be associated with genetic risk. Although DNA mutations are unequivocally linked to cancer, like fibrosis, only 5-10% of all cancers are linked to hereditary mutations (90). Specific somatic mutations arise in different tumor types, which has led to routine sequencing to improve personalized medicine. While some specific inhibitors have had modest effects at extending survival (91), the overall success rate is still poor across most cancers. Thus, the time has come to reevaluate cancer etiologies to refine prevention and treatment. In the subsequent portion of the review, we will investigate how key wound healing pathways cancer.

Some clues may address why a purely genetic approach has not sufficed. First, many known carcinogens are not mutagens (92, 93). Second, oncogenic mutations are routinely found in healthy tissues and benign tumors, accumulating with age in the absence of cancer (94–98). Third, some oncogenic mutations drive tumor growth in stem cells but have no effect in differentiated cells (99, 100). Recent studies point to environmental factors and tissue mechanics playing critical roles in tumor emergence and progression. Environmental factors like pollution, microplastics, and herbicides promote cancers without additional mutations in driver genes (10, 101–105) and reducing microenvironmental mechanical inputs or impairing mechanosensing impairs oncogene-mediated transformation and invasive behavior (106–109). Together, these findings show that mutations are not sufficient for tumor initiation and progression, suggesting the somatic mutation theory is too simplistic. Here, we consider how non-mutational oncogenic contributors (pollution, microplastics, obesity, infections) may trigger repeated episodes of wound healing and inflammation in a mutational landscape that instead of resolving leads to neoplasia and cell spread.

### Signaling

Signaling in wound healing is remarkably similar to cancer, often activating Receptor Tyrosine Kinases like the Epidermal Growth Factor Receptor (EGFR)-RAS-MAPK axis that lead to transcription of Immediate Early Genes like c-FOS or c-JUN, inhibition of epigenetic repressors like the Polycomb Group (PcG), and loss of H3K27me3 inhibitory epigenetic marks (11, 20). Nuclear cFOS and cJUN form the transcription factor AP1 which, in association with YAP/TAZ, drives transcription of genes essential for both wound healing and cancer (11, 109). Functionally, this is reflected in a temporary *lineage infidelity* that drive cell survival, plasticity, proliferation, and migration, which while it enables repopulation of the damaged tissue, becomes permanent in cancer (11, 110, 111). Remarkably, recent work showed that transient PcG inhibition is sufficient to drive AP1-dependent transcription, necessary for wound healing and tumorigenesis in the absence of mutations in *Drosophila* (103). Therefore, at least in some contexts, AP1-driven gene expression is sufficient to initiate cancer.

**Fig. 4:**
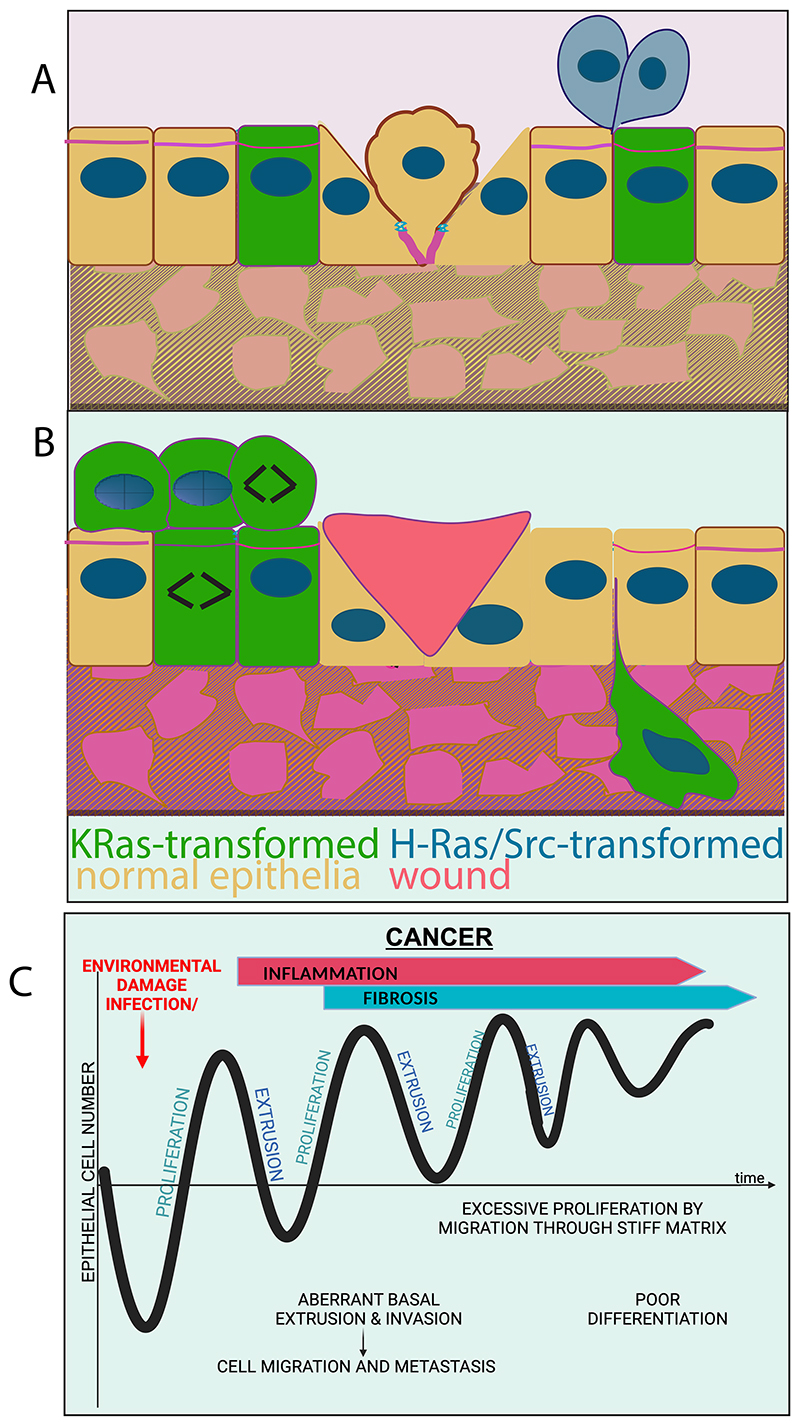
Defective wound response in cancer. (A, B) Schematics of oncogenically mutated cells with and without a wound response. In (A), some mutated cells may be effectively eliminated by Epithelial Defence Against Cancer (here, H-Ras or Src, blue cells), similar to how crowded, aged cells are eliminated by extrusion (center cell), whereas other cells with oncogenic mutations may accrue within the epithelium (here, KRas, green cells). However, when triggers like smoking, pollution, pathogen infections stimulate a wound healing response (B), oncogenically mutated epithelia (green) may be activated to proliferate without check (left) and extrude aberrantly basally, underneath the epithelium, enabling them to escape, dedifferentiate and metastasize (right). Here, tyrosine kinase inhibitors may target cells with amplified EGFR but not those under strain as they migrate through tight spaces during metastasis. (C) Schematic graph suggesting that wound healing in an oncogenic setting can initiate rounds of proliferation and defective extrusion that fail to return to homeostatic epithelial cell numbers, causing neoplastic properties, as well as fibrosis, inflammation, enhanced cell migration, and poor differentiation, known hallmarks of cancer.

In the last decade, YAP/TAZ have gained enormous attention in development, repair, fibrosis, and cancer, despite being dispensable for homeostatic cell turnover (111). During both wound healing and cancer, tissue and cell mechanics activate integrin-FAK and Rho signaling, leading to actomyosin-dependent nuclear YAP/TAZ accumulation. The resulting transcriptional changes enable wound healing and tumor growth, whereas inhibition of this axis prevents tissue repair, tumor growth, and metastasis (108, 112, 113). Mechanically activated ion channels Piezo1 and Piezo2 are key controllers of integrin-FAK, Rho, and YAP/TAZ signaling (108, 114). Moreover, it was recently shown that Piezo1 activates EGFR internalization and signaling in response to mechanical cues to promote a regenerative response involving AP1 and YAP that drives cycle reentry of differentiated cells within *ex vivo* mouse airways (115). Importantly, whereas canonical, ligand-triggered EGFR signaling requires EGFR tyrosine autophosphorylation, Piezo1-mediated mechanoresponses instead require EGFR serine phosphorylation via SRC and p38 kinases. These findings may account for chemoresistance to current therapeutic EGFR neutralizing antibodies and tyrosine kinase inhibitors, a common cause of cancer relapse and patient death (116) (Fig. 4A). Remarkably, separate recent work found skin injury activates EGFR signaling in wild type but not Ras-mutant cells, and the resulting differential proliferative boost acts as a tumor suppressing mechanism (117). Together, these findings advocate for considering context-dependent differences in signaling between homeostasis and repair/cancer when devising therapeutical strategies.

### Inflammation

As described above, inflammation is a key element of healthy wound healing and progressive fibrosis, but is also a well-established non-genomic cancer promoter triggered by environmental factors like air pollution, smoke, microplastics, viral infections, and surgery (1, 9, 10, 118–121). How inflammation awakens oncogenesis by pre-existing mutant cells is not completely understood. However, recent research identified interleukins (IL) released by macrophages during inflammation as key for cell reprogramming in oncogenesis. IL-6 and IL-33 released during pancreatitis trigger unique long-lasting epigenetic changes that facilitate tumorigenesis by KRAS-mutant pancreatic epithelial cells (119, 122). Importantly, this effect is seen even if KRAS mutations are induced after damage resolution, highlighting the long-lasting effects of even transient inflammation, suggesting an *epithelial memory* that accelerates barrier restoration upon subsequent damage but also facilitates autoimmune and oncogenic disorders (119, 123) (Fig. 4A). Other studies in zebrafish show that wounding adjacent to clones of pre-neoplastic skin cells leads to recruitment of immune cells and delivery of trophic prostaglandins that fuel cancer progression (124). Additionally, the persistent tissue wounding that Epidermolysis bullosa patients suffer due to very fragile skin triggers local inflammation that frequently results in development of squamous cell carcinomas (125). In humans, IL-1β release after exposure to air pollutants reprograms lung epithelial cells with pre-existing EGFR mutations into a progenitor-like state with active JAK-STAT signaling and tumor growth (10). Hence, cell identity is not fixed and instead results from integrating genetics and epigenetics with environmental and microenvironmental alterations to drive oncogenesis. In this way, we propose that wild-type wound healing responses to environmental insults and resulting inflammation could cause continuous feed-forward cycles of proliferation and metaplasia in mutant cells (Fig. 4B).

### Epithelial cell population control

Besides cell identity, inflammation alters interactions between epithelial cells. The patchwork of clones comprising adult epithelia compete for limited space, nutrients, and survival cues (126). Somatic mutations in cancer associated genes (e.g., Myc, EGFR, Ras, p53, or JAK-STAT components) alter competitive fitness (127). Competition-based mutant cell (loser) elimination by wild type neighbors (winners) is an innate tumor-suppressing mechanism frequently known as *Epithelial Defense Against Cancer* (EDAC) (126, 128). However, competition is context-dependent, and mutation combinations or environmental inputs like a high-fat diet, hyperinsulinemia, chronic inflammation, or fibrosis upend competition roles, facilitating mutant clone expansion at the expense of wild type cell elimination (9, 126, 129–133). This inversion may contribute to the links between diet, obesity, diabetes, inflammation, and cancer.

Apoptosis plays essential roles in *loser* cell elimination during classical competition models in *Drosophila* (127). However, apoptosis is dispensable for mammalian cell competition and extrusion, the essential cell elimination mechanism balancing cell proliferation and migration (59, 131). Moreover, during wound closure, mechanical p53 activation sensitizes leader migrating cells to crowding, facilitating their elimination by apoptosis or crowding-induced extrusion after wound closure (134, 135). Activated p53 also drives extrusion after replicative stress, even in the absence of apoptosis in *C. elegans* and mammals (136). Thus, repairing tissues could similarly re-balance differentiated cell populations by extruding poorly differentiated or DNA-damaged cells that emerge during initial repair phases requiring rapid division. Extrusion defects could result in the accumulation of undifferentiated or mutant cells, both cancer hallmarks, and also contribute to poor barrier function that would promote inflammation (137). Thus, extrusion is likely to play a vital role in wound resolution and tissue refinement following wound healing (Fig. 4B)

Although extrusion can eliminate potentially cancerous cells, mutations associated with aggressive cancers (e.g. KRas, APC) can also hijack extrusion to instead promote cell invasion (137, 138) (Fig. 4A). Additionally, as basally extruding cells invade, they pinch off their apical epithelial determinants, causing them to partially dedifferentiate and endow new plasticity (139), akin to partial EMT during wound healing (see earlier). Invading cells migrate along tortuous confining environments that deform their nuclei, promoting mesenchymal gene expression, which enhances their survival into large internal cell masses (140). Additionally, the mechanical pressure migrating cells experience likely enhances their proliferation and chemoresistance via the Piezo1-EGFR pathway previously mentioned (115). These findings may explain why metastatic cells become poorly differentiated and chemo-resistant and why stiff environments contribute to worse prognoses. When considering wounding/damage as a cancer stimulus, defects in extrusion in the backdrop of these mutations could promote not only accumulation of damaged proliferating cells, but also, and likely more importantly, the invasion of cells that contribute to the major life-threatening complication of cancer, metastasis (Fig. 4A).

### Angiogenesis

New blood vessel formation, termed angiogenesis, is another big parallel between wound healing and cancer. Importantly, local environment rather than mutations control angiogenesis. Oxygen and nutrients are limiting for both repairing tissues and oncogenic growth. In both scenarios, vessel sprouting in response to locally released angiogenic factors, like vascular endothelial growth factor (VEGF) and thrombospondin, which generates loosely organized networks of leaky capillaries. However, newly sprouting vessels mature in healthy wound healing but not so during tumorigenesis (38), resulting in disrupted blood flow and interstitial liquid accumulation, which, in turn, promotes malignant Piezo1-YAP/TAZ mechanosignalling (111). Inflammation also plays key roles in angiogenesis, with Langerhans cells promoting endothelial cell proliferation and migration and macrophages being key for angiogenesis resolution (38, 141). Notably, unresolved angiogenesis is common to cancer, benign tumors, and keloid scars arising from dysfunctional skin wound healing (142). Thus, a controlled inflammatory response is essential for terminating angiogenesis during tissue repair to avoid excessive cell proliferation.

## Conclusions

Organ fibrosis, cancer, and chronic inflammation contribute to over half of all mortality (143). Therefore, novel therapies addressing these common mechanisms could revolutionise public health. Many similarities exist across all these diseases, especially when considering faulty wound healing responses. Yet, it is not clear why some wounding responses lead to inflammation in some instances and fibrosis or cancer in others. Often these responses are linked, since both liver and lung fibrosis increase the risk of hepatocellular carcinoma (144) and lung cancer (145), suggesting that dysregulated wound healing may be the critical initiating step in both processes. A large study found that high neutrophil count and inflammatory markers predicted all-cause mortality over 8 years (146), further supporting this hypothesis. It is possible that these wound responses could proceed in a step-wise fashion, with chronic inflammation giving rise to fibrosis, which in turn, gives rise to cancer. Alternatively, wound healing may underlie each, with random chance or different genetic, inflammatory, or biophysical backdrops determining how an aged tissue responds. Therefore, strategies that correct the initial wound healing step could prevent a multitude of diseases. Understanding the genetic and sequential environmental triggers that ignite these common, yet deadly, diseases will be key to developing new approaches to preventing and treating them.

Recognizing that these diseases are ignited not only by genes but by the environment causing micro-wounds is a double-edged sword. Our ability to control our environmental exposure to pollution, microplastics, smoke, age, and viruses may be limited, but understanding how these signals fuel disease may enable us to design better ways to treat or better yet prevent them. For instance, identifying signals activated only in wound environments, such as Piezo1-induced EGFR signaling may provide better targets for cancer (115). On the other hand, public health policies aimed at reducing air pollutants and microplastics (54, 147) could play a crucial role in disease prevention, as witnessed for smoking cessation (148). Identifying agents within our food chain that contribute to the obesity epidemic would greatly impact most diseases (149). Given that prevention measures impact 80% of human cancers and fibrogenesis, preventive public health policies are essential to reduce overall disease burden (150).

Here, we lay out a perspective on diseases that invokes the cumulative damage that individuals accrue to account for many of the pathologies and diseases that typically arise with age. Our discussion is not meant to minimise the role of mutations in driving disease but to bring to the foreground the random and continuous battles we encounter as we make our way through life. We readily acknowledge that our graphs and ideas are overly simplistic. Yet, rather than thinking about disease as a collection of symptoms, we hope to highlight disease aetiology by thinking about it chronologically. By understanding triggers that launch a tissue repair process into a devolving disease, we may be better armed to prevent or treat a variety of pathologies.

## References

[1] Virchow R (1863). Die Krankenaften Geschwulste.

[2] Haddow A (1973). Advances in Cancer Research.

[3] Flier JS, Underhill LH, Dvorak HF (1986). Tumors: Wounds That Do Not Heal. New England Journal of Medicine.

[4] Martin D, Gutkind JS (2008). Human tumor-associated viruses and new insights into the molecular mechanisms of cancer. Oncogene.

[5] Bister K (2015). Discovery of oncogenes: The advent of molecular cancer research. Proc Natl Acad Sci U S A.

[6] Jassim A, Rahrmann EP, Simons BD, Gilbertson RJ (2023). Cancers make their own luck: theories of cancer origins. Nature Reviews Cancer.

[7] Swanton C (2024). Embracing cancer complexity: Hallmarks of systemic disease. Cell.

[8] Rahal Z, Scheet P, Kadara H (2024). Somatic Mutations in Normal Tissues: Calm before the Storm. Cancer Discovery.

[9] Weeden CE, Hill W, Lim EL, Grönroos E, Swanton C (2023). Impact of risk factors on early cancer evolution. Cell.

[10] Hill W (2023). Lung adenocarcinoma promotion by air pollutants. Nature.

[11] MacCarthy-Morrogh L, Martin P (2020). The hallmarks of cancer are also the hallmarks of wound healing. Sci Signal.

[12] Peña OA, Martin P (2024). Cellular and molecular mechanisms of skin wound healing. Nature Reviews Molecular Cell Biology.

[13] Headland SE, Norling LV (2015). The resolution of inflammation: Principles and challenges. Semin Immunol.

[14] Soliman AM, Barreda DR (2023). Acute Inflammation in Tissue Healing. International Journal of Molecular Sciences.

[15] Chen WY, Rogers AA (2007). Recent insights into the causes of chronic leg ulceration in venous diseases and implications on other types of chronic wounds. Wound Repair Regen.

[16] Sugimoto MA, Sousa LP, Pinho V, Perretti M, Teixeira MM (2016). Resolution of Inflammation: What Controls Its Onset?. Front Immunol.

[17] Aragona M (2017). Defining stem cell dynamics and migration during wound healing in mouse skin epidermis. Nat Commun.

[18] Park S (2017). Tissue-scale coordination of cellular behaviour promotes epidermal wound repair in live mice. Nat Cell Biol.

[19] Turley J (2024). Deep learning reveals a damage signalling hierarchy that coordinates different cell behaviours driving wound re-epithelialisation. Development.

[20] Shaw T, Martin P (2009). Epigenetic reprogramming during wound healing: loss of polycomb-mediated silencing may enable upregulation of repair genes. EMBO Rep.

[21] Weavers H, Wood W, Martin P (2019). Injury Activates a Dynamic Cytoprotective Network to Confer Stress Resilience and Drive Repair. Curr Biol.

[22] Nunan R (2015). Ephrin-Bs Drive Junctional Downregulation and Actin Stress Fiber Disassembly to Enable Wound Re-epithelialization. Cell Rep.

[23] Rohani MG, Parks WC (2015). Matrix remodeling by MMPs during wound repair. Matrix Biol.

[24] Swaney MH, Kalan LR (2021). Living in Your Skin: Microbes, Molecules, and Mechanisms. Infect Immun.

[25] De Lorenzo G, Ferrari S, Cervone F, Okun E (2018). Extracellular DAMPs in Plants and Mammals: Immunity, Tissue Damage and Repair. Trends Immunol.

[26] Liu L (2019). Induction of neutrophil extracellular traps during tissue injury: Involvement of STING and Toll-like receptor 9 pathways. Cell Prolif.

[27] Willenborg S (2021). Mitochondrial metabolism coordinates stage-specific repair processes in macrophages during wound healing. Cell Metab.

[28] Shook BA (2018). Myofibroblast proliferation and heterogeneity are supported by macrophages during skin repair. Science.

[29] Mori R, Shaw TJ, Martin P (2008). Molecular mechanisms linking wound inflammation and fibrosis: knockdown of osteopontin leads to rapid repair and reduced scarring. J Exp Med.

[30] Knipper JA (2015). Interleukin-4 Receptor α Signaling in Myeloid Cells Controls Collagen Fibril Assembly in Skin Repair. Immunity.

[31] Hinz B (2015). The extracellular matrix and transforming growth factor-β1: Tale of a strained relationship. Matrix Biol.

[32] Ferguson MW, O’Kane S (2004). Scar-free healing: from embryonic mechanisms to adult therapeutic intervention. Philos Trans R Soc Lond B Biol Sci.

[33] Occleston NL, Laverty HG, O’Kane S, Ferguson MW (2008). Prevention and reduction of scarring in the skin by Transforming Growth Factor beta 3 (TGFbeta3): from laboratory discovery to clinical pharmaceutical. J Biomater Sci Polym Ed.

[34] Jiang D, Rinkevich Y (2021). Distinct fibroblasts in scars and regeneration. Curr Opin Genet Dev.

[35] Usansky I (2021). A developmental basis for the anatomical diversity of dermis in homeostasis and wound repair. J Pathol.

[36] Szpaderska AM, Zuckerman JD, DiPietro LA (2003). Differential injury responses in oral mucosal and cutaneous wounds. J Dent Res.

[37] Cho H (2024). In the face and neck, keloid scar distribution is related to skin thickness and stiffness changes associated with movement. Wound Repair Regen.

[38] Gurevich DB (2018). Live imaging of wound angiogenesis reveals macrophage orchestrated vessel sprouting and regression. Embo j.

[39] Lucas T (2010). Differential roles of macrophages in diverse phases of skin repair. J Immunol.

[40] Komi DEA, Khomtchouk K, Santa Maria PL (2020). A Review of the Contribution of Mast Cells in Wound Healing: Involved Molecular and Cellular Mechanisms. Clin Rev Allergy Immunol.

[41] Witherden DA, Havran WL (2013). Cross-talk between intraepithelial γδ T cells and epithelial cells. J Leukoc Biol.

[42] Khalid KA, Nawi AFM, Zulkifli N, Barkat MA, Hadi H (2022). Aging and Wound Healing of the Skin: A Review of Clinical and Pathophysiological Hallmarks. Life (Basel).

[43] Wick KD, Ware LB, Matthay MA (2024). Acute respiratory distress syndrome. BMJ.

[44] Stewart I (2022). Residual Lung Abnormalities after COVID-19 Hospitalization: Interim Analysis of the UKILD Post–COVID-19 Study. American Journal of Respiratory and Critical Care Medicine.

[45] Liang J (2023). Reciprocal interactions between alveolar progenitor dysfunction and aging promote lung fibrosis. eLife.

[46] Bartleson JM (2021). SARS-CoV-2, COVID-19 and the aging immune system. Nature Aging.

[47] Han S, Budinger GRS, Gottardi CJ (2023). Alveolar epithelial regeneration in the aging lung. The Journal of Clinical Investigation.

[48] Parimon T (2023). Senescence of alveolar epithelial progenitor cells: a critical driver of lung fibrosis. American Journal of Physiology-Cell Physiology.

[49] Margaritte-Jeannin P (2004). HLA-DQ relative risks for coeliac disease in European populations: a study of the European Genetics Cluster on Coeliac Disease. Tissue Antigens.

[50] Brodin P (2015). Variation in the Human Immune System Is Largely Driven by Non-Heritable Influences. Cell.

[51] Furman D (2019). Chronic inflammation in the etiology of disease across the life span. Nature Medicine.

[52] Ali N (2024). The potential impacts of micro-and-nano plastics on various organ systems in humans. eBioMedicine.

[53] Ehlers S, Kaufmann SHE (2010). Infection, inflammation, and chronic diseases: consequences of a modern lifestyle. Trends in Immunology.

[54] Thompson RC (2024). Twenty years of microplastic pollution research—what have we learned?. Science.

[55] Constant DA, Nice TJ, Rauch I (2021). Innate immune sensing by epithelial barriers. Curr Opin Immunol.

[56] Rosenblatt J, Raff MC, Cramer LP (2001). An epithelial cell destined for apoptosis signals its neighbors to extrude it by an actin- and myosin-dependent mechanism. Curr Biol.

[57] Liesman RM (2014). RSV-encoded NS2 promotes epithelial cell shedding and distal airway obstruction. The Journal of Clinical Investigation.

[58] Moshiri J, Craven AR, Mixon SB, Amieva MR, Kirkegaard K (2023). Mechanosensitive extrusion of Enterovirus A71-infected cells from colonic organoids. Nat Microbiol.

[59] Eisenhoffer GT (2012). Crowding induces live cell extrusion to maintain homeostatic cell numbers in epithelia. Nature.

[60] Kajita M, Fujita Y (2015). EDAC: Epithelial defence against cancer-cell competition between normal and transformed epithelial cells in mammals. J Biochem.

[61] Gudipaty SA (2017). Mechanical stretch triggers rapid epithelial cell division through Piezo1. Nature.

[62] Bagley DC (2024). Bronchoconstriction damages airway epithelia by crowding-induced excess cell extrusion. Science.

[63] Christenson SA, Smith BM, Bafadhel M, Putcha N (2022). Chronic obstructive pulmonary disease. The Lancet.

[64] Perotin J-M (2014). Delay of airway epithelial wound repair in COPD is associated with airflow obstruction severity. Respiratory Research.

[65] Walters EH, Shukla SD, Mahmood MQ, Ward C (2021). Fully integrating pathophysiological insights in COPD: an updated working disease model to broaden therapeutic vision. European Respiratory Review.

[66] Bouin M (2002). Rectal distention testing in patients with irritable bowel syndrome: sensitivity, specificity, and predictive values of pain sensory thresholds. Gastroenterology.

[67] Coates MD (2023). Abdominal Pain in Inflammatory Bowel Disease: An Evidence-Based, Multidisciplinary Review. Crohns Colitis 360.

[68] Ravimohan S, Kornfeld H, Weissman D, Bisson GP (2018). Tuberculosis and lung damage: from epidemiology to pathophysiology. Eur Respir Rev.

[69] Mathai SK, Newton CA, Schwartz DA, Garcia CK (2016). Pulmonary fibrosis in the era of stratified medicine. Thorax.

[70] Leavy OC (2023). The Use of Genetic Information to Define Idiopathic Pulmonary Fibrosis in UK Biobank. Chest.

[71] Peters A (2023). Ambient air pollution and Alzheimer’s disease: the role of the composition of fine particles. Proceedings of the National Academy of Sciences.

[72] Fadista J (2021). Shared genetic etiology between idiopathic pulmonary fibrosis and COVID-19 severity. EBioMedicine.

[73] Verma A (2022). A MUC5B Gene Polymorphism, rs35705950-T, Confers Protective Effects Against COVID-19 Hospitalization but Not Severe Disease or Mortality. Am J Respir Crit Care Med.

[74] Costain G (2022). Hereditary Mucin Deficiency Caused by Biallelic Loss of Function of MUC5B. Am J Respir Crit Care Med.

[75] Oldham JM (2023). PCSK6 and Survival in Idiopathic Pulmonary Fibrosis. American Journal of Respiratory and Critical Care Medicine.

[76] Zhou H (2023). UBQLN1 deficiency mediates telomere shortening and IPF through interacting with RPA1. PLOS Genetics.

[77] Fainberg HP (2024). Cluster analysis of blood biomarkers to identify molecular patterns in pulmonary fibrosis: assessment of a multicentre, prospective, observational cohort with independent validation. The Lancet Respiratory Medicine.

[78] Khanna D (2022). Systemic Sclerosis-Associated Interstitial Lung Disease: How to Incorporate Two Food and Drug Administration-Approved Therapies in Clinical Practice. Arthritis Rheumatol.

[79] Bialik JF (2019). Profibrotic epithelial phenotype: a central role for MRTF and TAZ. Scientific reports.

[80] Ma HY (2023). Inhibition of MRTF activation as a clinically achievable anti-fibrotic mechanism for pirfenidone. Eur Respir J.

[81] Kulkarni AB (1993). Transforming growth factor beta 1 null mutation in mice causes excessive inflammatory response and early death. Proc Natl Acad Sci U S A.

[82] Massagué J (2008). TGFbeta in Cancer. Cell.

[83] John AE (2016). Loss of epithelial Gq and G11 signaling inhibits TGFβ production but promotes IL-33-mediated macrophage polarization and emphysema. Sci Signal.

[84] Froese AR (2016). Stretch-induced Activation of Transforming Growth Factor-β1 in Pulmonary Fibrosis. Am J Respir Crit Care Med.

[85] Tatler AL (2011). Integrin αvβ5-mediated TGF-β activation by airway smooth muscle cells in asthma. J Immunol.

[86] Kim KK (2009). Epithelial cell α3β1 integrin links β-catenin and Smad signaling to promote myofibroblast formation and pulmonary fibrosis. The Journal of Clinical Investigation.

[87] Goodwin AT (2023). Stretch regulates alveologenesis and homeostasis via mesenchymal Gαq/11-mediated TGFβ2 activation. Development.

[88] Allen RJ (2017). Genetic variants associated with susceptibility to idiopathic pulmonary fibrosis in people of European ancestry: a genome-wide association study. Lancet Respir Med.

[89] Cairns JT (2020). Loss of ELK1 has differential effects on age-dependent organ fibrosis. Int J Biochem Cell Biol.

[90] Nagy R, Sweet K, Eng C (2004). Highly penetrant hereditary cancer syndromes. Oncogene.

[91] Morgillo F, Della Corte CM, Fasano M, Ciardiello F (2016). Mechanisms of resistance to EGFR-targeted drugs: lung cancer. ESMO Open.

[92] Kucab JE (2019). A Compendium of Mutational Signatures of Environmental Agents. Cell.

[93] Riva L (2020). The mutational signature profile of known and suspected human carcinogens in mice. Nature Genetics.

[94] Martincorena I (2018). Somatic mutant clones colonize the human esophagus with age. Science.

[95] Pollock PM (2003). High frequency of BRAF mutations in nevi. Nature Genetics.

[96] Rahal Z, Sinjab A, Wistuba II, Kadara H (2022). Game of clones: Battles in the field of carcinogenesis. Pharmacology & Therapeutics.

[97] Yizhak K (2019). RNA sequence analysis reveals macroscopic somatic clonal expansion across normal tissues. Science.

[98] Yokoyama A (2019). Age-related remodelling of oesophageal epithelia by mutated cancer drivers. Nature.

[99] Barker N (2009). Crypt stem cells as the cells-of-origin of intestinal cancer. Nature.

[100] Zhu M (2019). Somatic Mutations Increase Hepatic Clonal Fitness and Regeneration in Chronic Liver Disease. Cell.

[101] Casasent AK (2022). Learning to distinguish progressive and non-progressive ductal carcinoma in situ. Nature Reviews Cancer.

[102] Lips EH (2022). Genomic analysis defines clonal relationships of ductal carcinoma in situ and recurrent invasive breast cancer. Nature Genetics.

[103] Parreno V (2024). Transient loss of Polycomb components induces an epigenetic cancer fate. Nature.

[104] Chang VC (2023). Glyphosate exposure and urinary oxidative stress biomarkers in the Agricultural Health Study. JNCI: Journal of the National Cancer Institute.

[105] Li S, Keenan JI, Shaw IC, Frizelle FA (2023). Could Microplastics Be a Driver for Early Onset Colorectal Cancer?. Cancers (Basel).

[106] Cordenonsi M (2011). The Hippo Transducer TAZ Confers Cancer Stem Cell-Related Traits on Breast Cancer Cells. Cell.

[107] Panciera T (2020). Reprogramming normal cells into tumour precursors requires ECM stiffness and oncogene-mediated changes of cell mechanical properties. Nature materials.

[108] Pardo-Pastor C (2018). Piezo2 channel regulates RhoA and actin cytoskeleton to promote cell mechanobiological responses. Proceedings of the National Academy of Sciences.

[109] Zanconato F (2015). Genome-wide association between YAP/TAZ/TEAD and AP-1 at enhancers drives oncogenic growth. Nature Cell Biology.

[110] Ge Y (2017). Stem Cell Lineage Infidelity Drives Wound Repair and Cancer. Cell.

[111] Piccolo S, Panciera T, Contessotto P, Cordenonsi M (2023). YAP/TAZ as master regulators in cancer: modulation, function and therapeutic approaches. Nat Cancer.

[112] Grzelak EM (2023). Pharmacological YAP activation promotes regenerative repair of cutaneous wounds. Proceedings of the National Academy of Sciences.

[113] Thompson BJ (2020). YAP/TAZ: Drivers of Tumor Growth, Metastasis, and Resistance to Therapy. BioEssays.

[114] Hung W-C (2016). Confinement Sensing and Signal Optimization via Piezo1/PKA and Myosin II Pathways. Cell Reports.

[115] Pardo-Pastor C, Rosenblatt J (2023). Piezo1 activates non-canonical EGFR endocytosis and signaling. Sci Adv.

[116] Thomas R, Weihua Z (2019). Rethink of EGFR in Cancer With Its Kinase Independent Function on Board. Front Oncol.

[117] Gallini S (2023). Injury prevents Ras mutant cell expansion in mosaic skin. Nature.

[118] Alonso-Curbelo D (2021). A gene–environment-induced epigenetic program initiates tumorigenesis. Nature.

[119] Del Poggetto E (2021). Epithelial memory of inflammation limits tissue damage while promoting pancreatic tumorigenesis. Science.

[120] Park JH (2023). Polypropylene microplastics promote metastatic features in human breast cancer. Scientific reports.

[121] Bogden AE, Moreau JP, Eden PA (1997). Proliferative response of human and animal tumours to surgical wounding of normal tissues: onset, duration and inhibition. Br J Cancer.

[122] Burdziak C (2023). Epigenetic plasticity cooperates with cell-cell interactions to direct pancreatic tumorigenesis. Science.

[123] Naik S (2017). Inflammatory memory sensitizes skin epithelial stem cells to tissue damage. Nature.

[124] Antonio N (2015). The wound inflammatory response exacerbates growth of pre-neoplastic cells and progression to cancer. Embo j.

[125] Bonamonte D (2022). Squamous Cell Carcinoma in Patients with Inherited Epidermolysis Bullosa: Review of Current Literature. Cells.

[126] van Neerven SM, Vermeulen L (2023). Cell competition in development, homeostasis and cancer. Nature Reviews Molecular Cell Biology.

[127] Morata G (2021). Cell competition: A historical perspective. Dev Biol.

[128] Tanimura N, Fujita Y (2020). Epithelial defense against cancer (EDAC). Seminars in Cancer Biology.

[129] Kohashi K (2021). Sequential oncogenic mutations influence cell competition. Current Biology.

[130] Pothapragada SP, Gupta P, Mukherjee S, Das T (2022). Matrix mechanics regulates epithelial defence against cancer by tuning dynamic localization of filamin. Nature Communications.

[131] Colom B (2021). Mutant clones in normal epithelium outcompete and eliminate emerging tumours. Nature.

[132] Sanaki Y, Nagata R, Kizawa D, Léopold P, Igaki T (2020). Hyperinsulinemia Drives Epithelial Tumorigenesis by Abrogating Cell Competition. Developmental Cell.

[133] Sasaki A (2018). Obesity Suppresses Cell-Competition-Mediated Apical Elimination of RasV12-Transformed Cells from Epithelial Tissues. Cell Reports.

[134] Wagstaff L (2016). Mechanical cell competition kills cells via induction of lethal p53 levels. Nature Communications.

[135] Kozyrska K (2022). p53 directs leader cell behavior, migration, and clearance during epithelial repair. Science.

[136] Dwivedi VK (2021). Replication stress promotes cell elimination by extrusion. Nature.

[137] Slattum GM, Rosenblatt J (2014). Tumour cell invasion: an emerging role for basal epithelial cell extrusion. Nature Reviews Cancer.

[138] Fadul J, Rosenblatt J (2018). The forces and fates of extruding cells. Curr Opin Cell Biol.

[139] Fadul J (2021). KRas-transformed epithelia cells invade and partially dedifferentiate by basal cell extrusion. Nature Communications.

[140] Zulueta-Coarasa T, Fadul J, Ahmed M, Rosenblatt J (2022). Physical confinement promotes mesenchymal trans-differentiation of invading transformed cells in vivo. iScience.

[141] Lewis JM (2015). Langerhans Cells Facilitate UVB-Induced Epidermal Carcinogenesis. J Invest Dermatol.

[142] Zhang M, Chen H, Qian H, Wang C (2023). Characterization of the skin keloid microenvironment. Cell Commun Signal.

[143] Massen GM (2024). Using Routinely Collected Electronic Healthcare Record Data to Investigate Fibrotic Multimorbidity in England. Clin Epidemiol.

[144] Younossi Z (2019). Nonalcoholic Steatohepatitis Is the Fastest Growing Cause of Hepatocellular Carcinoma in Liver Transplant Candidates. Clin Gastroenterol Hepatol.

[145] Hubbard R, Venn A, Lewis S, Britton J (2000). Lung cancer and cryptogenic fibrosing alveolitis. A population-based cohort study. Am J Respir Crit Care Med.

[146] Proctor MJ (2015). Systemic inflammation predicts all-cause mortality: a glasgow inflammation outcome study. PLoS One.

[147] Brown E, MacDonald A, Allen S, Allen D (2023). The potential for a plastic recycling facility to release microplastic pollution and possible filtration remediation effectiveness. Journal of Hazardous Materials Advances.

[148] Murray RL, O’Dowd E (2023). Smoking cessation and lung cancer: never too late to quit. The Lancet Public Health.

[149] Ren X-M, Kuo Y, Blumberg B (2020). Agrochemicals and obesity. Molecular and Cellular Endocrinology.

[150] Tran KB (2022). The global burden of cancer attributable to risk factors, 2010–19: a systematic analysis for the Global Burden of Disease Study 2019. The Lancet.

